# Impact of oxidative stress on *Magnetospirillum gryphiswaldense* MSR-1 physiology and magnetosome biomineralization at the single-cell level

**DOI:** 10.1128/mbio.03265-25

**Published:** 2025-12-29

**Authors:** Marta Masó-Martínez, Julika Radecke, Craig MacGregor-Chatwin, Paul D. Topham, Alfred Fernández-Castané

**Affiliations:** 1 Energy and Bioproducts Research Institute, Aston University 1722, Birmingham, United Kingdom; 2 Aston Institute for Membrane Excellence, Aston University 1722, Birmingham, United Kingdom; 3 Electron Bio-Imaging Centre (eBIC), Diamond Light Source, Harwell Science and Innovation Campus 70597 , Didcot, United Kingdom; The University of Texas Health Science Center at Houston, Houston, Texas, USA

**Keywords:** magnetotactic bacteria, magnetosomes, biomineralization, correlative microscopy, cryo-electron tomography, oxidative stress

## Abstract

Magnetotactic bacteria, such as *Magnetospirillum gryphiswaldense* MSR-1, naturally produce magnetosomes—intracellular magnetic nanoparticles that enable navigation within geomagnetic fields. Magnetosomes hold significant potential for biomedical and biotechnological applications; however, key aspects of their biomineralization remain poorly understood. This study investigates how oxidative stress, induced by hydrogen peroxide and iron, influences magnetosome formation and bacterial physiology under aerobic and microaerobic conditions. Single-cell advanced microscopy and high-throughput techniques revealed that microaerobic conditions supported robust magnetosome production and larger magnetite crystals while maintaining low oxidative stress levels. In contrast, aerobic conditions suppressed magnetosome formation, reduced intracellular iron content, and increased reactive oxygen species (ROS) levels. High extracellular iron enhanced the formation of longer magnetosome chains in microaerobic cultures without causing toxicity but reduced cell viability under aerobic conditions. Hydrogen peroxide exposure caused mild damage and a 25% viability drop in magnetosome-producing cells but led to severe damage and an 80% viability drop in non-magnetosome-producing cells, along with chain fragmentation and smaller magnetite crystals. These results suggest that magnetosome-producing cells exhibit greater resilience to oxidative stress, potentially due to ROS scavenging properties of magnetosomes, and highlight the intricate interplay between oxidative stress, iron regulation, and magnetosome biomineralization. Single-cell analysis revealed heterogeneity in physiological responses, further demonstrating the complexity of these processes. These findings underscore the importance of monitoring physiological changes during production processes to enhance the efficiency and robustness of magnetosome synthesis. The insights gained provide a foundation for improving bioprocesses for large-scale production of high-quality magnetosomes, advancing their applications in biomedicine and biotechnology.

**IMPORTANCE:**

Magnetotactic bacteria (MTB) are fascinating microorganisms with the capacity to produce iron oxide nanomagnets, namely magnetosomes. These nanomagnets passively align with magnetic fields, providing the basis for magnetic guidance when coupled with bacterial motility. This unique feature has inspired the concept of MTB as a self-propelled medical device to deliver therapeutic cargo. To this end, survival in physiological conditions and other microenvironments is essential. This study reveals the interplay between MTB physiology and magnetosome biomineralization subject to oxidative stress and varying environmental conditions, employing a holistic multi-parametric approach. Our results provide an in-depth understanding of the metabolic and physiological mechanisms at the single-cell level. This is crucial to develop more robust MTB biomanufacturing strategies to produce bacteria that possess the required quality attributes, thus paving the way for future biotechnological and biomedical application studies.

## INTRODUCTION

Oxidative stress is a consequence of the excessive production of reactive oxygen species (ROS), which are the by-products of oxygen reduction in metabolic processes (1). Organisms residing in oxygen-rich environments have developed scavenging systems to mitigate this oxidative stress (2). However, when ROS accumulation surpasses the cellular antioxidant defense mechanisms, it can lead to damage to nucleic acids, proteins, lipids, and other cellular components, directly compromising cell viability (3). Controlling ROS accumulation is thus a critical parameter in bioprocessing. Excessive ROS not only affects cell health and viability but also induces oxidative modifications that can affect product quality, introduce variability in cell growth, and limit productivity. Therefore, the activation of cellular stress responses that alter metabolism and cell behavior can further impact the efficiency and consistency of the bioprocess.

The magnetic nanoparticles naturally produced by magnetotactic bacteria (MTB), called magnetosomes, represent an attractive alternative to chemically synthesized magnetic nanoparticles for biomedical, biotechnological, and environmental applications due to their unique properties (4). Magnetosomes are intracellular membrane-enveloped magnetic nanosized crystals of either magnetite (Fe_3_O_4_) or greigite (Fe_3_S_4_), depending on the species, typically arranged in a needle-like chain that acts as a compass needle, enabling an active orientation within the Earth’s magnetic field. These properties make magnetosomes and their producing bacteria attractive for applications such as magnetic hyperthermia, theranostic devices, magnetic resonance imaging contrast agents, and bioremediation (5–8). Their uniform size, high crystallinity, and biocompatibility further enhance their potential for such applications, highlighting the relevance of studying their biosynthesis and cellular regulation.

Magnetosome synthesis involves a complex and highly regulated biomineralization process that requires large amounts of iron and optimal microaerobic conditions, typically around 25 mbar O_2_ (approximately 0.1%–1% O_2_) (9). However, elevated intracellular levels of iron ions can lead to significant oxidative stress as hydrogen peroxide (H_2_O_2_) reacts with ferrous iron (Fe^2+^) in the so-called “Fenton reaction” to produce hydroxyl radicals (OH•) (10), which are among the most toxic ROS. Therefore, monitoring cell physiology during cell growth and magnetosome production is crucial to ensure cell viability and to control iron homeostasis to avoid the formation of specific physiological inhibitors, such as excessive ROS accumulation.

The mechanisms by which MTB tackle oxidative stress remain a significant and unresolved research question. Several studies have identified oxidative stress regulation mechanisms in these bacteria that are similar to those in other prokaryotic organisms. For instance, the presence of an OxyR-like redox-sensing transcription factor has been identified in some MTB species, such as *Magnetospirillum gryphiswaldense* MSR-1 and *Magnetospirillum magneticum* AMB-1 (11–13). Additionally, it has been observed that magnetosomes possess peroxidase-like activity, which could contribute to lowering overall ROS levels (14). This potential ROS-scavenging role of magnetosomes points to additional functions for magnetosome chains beyond their role in magnetic orientation.

In this study, we investigated the physiological responses of the MTB species *M. gryphiswaldense* MSR-1 to sudden oxidative perturbations to elucidate how cells cope with acute stress conditions. We hypothesized that magnetosome-producing bacteria would exhibit greater tolerance to oxidative challenges than non-producing bacteria, owing to the potential ROS-scavenging role of magnetosomes. To test this, MSR-1 cultures were exposed to H_₂_O_₂_ and high levels of extracellular iron (in the form of Fe(III)-citrate) while monitoring key physiological parameters, including cell viability, cell growth, intracellular iron accumulation, polyhydroxyalkanoate (PHA) granule formation, and ROS accumulation.

A combination of complementary analytical techniques was applied to capture responses across scales. Flow cytometry (FCM) was employed as an in-line tool for the rapid assessment of the physiological state of MSR-1 cells, focusing on parameters such as cell viability, intracellular ROS and iron levels, and PHA formation; inductively coupled plasma optical emission spectroscopy (ICP-OES) was used as an offline tool to quantify magnetosome and iron content; correlative light and electron microscopy (CLEM) combined cryo-electron and cryo-fluorescence imaging to simultaneously visualize magnetosomes, ROS, and intracellular iron at the single-cell level. To the best of our knowledge, only a few correlative microscopy studies have been performed on MTB (15–17), but this represents the first to combine these methods in MTB, providing an integrated framework to investigate the relationship between ROS, the intracellular labile iron pool, and magnetosome content at the single-cell level. By integrating high-resolution and high-throughput techniques within a single experimental design, this study offers mechanistic insight into how MTB cope with acute oxidative stress and lays the groundwork for optimizing magnetosome and MTB biomanufacturing platforms.

## MATERIALS AND METHODS

### Strains, growth media, and culture conditions

*M. gryphiswaldense* MSR-1 (DMSZ 6631) was grown in flask standard medium (FSM), comprising 3.5 g·L^−1^ potassium L-Lactate, 0.1 g·L^−1^ KH_2_PO_4_, 0.15 g·L^−1^ MgSO_4_·7H_2_O, 2.38 g·L^−1^ HEPES, 0.34 g·L−1 NaNO_3_, 0.1 g·L^−1^ yeast extract, 3 g·L^−1^ soy bean peptone, 100 μM iron citrate (C_6_H_5_FeO_7_), and 5 mL·L^−1^ EDTA-chelated trace elements solution (EDTA-TES). EDTA-TES solution consisted of 5.2 g·L^−1^ EDTA disodium salt; 30 mg·L^−1^ H_3_BO_3_; 85.4 mg·L^−1^ MnSO_4_·H_2_O; 190 mg·L^−1^; 2.1 g·L^−1^ FeSO_4_·7H_2_O, CoCl_2_ g·L^−1^; 4 mg·L^−1^ NiCl_2_·6H_2_O; 2 mg·L^−1^ CuCl_2_·2H_2_O; 44 mg·L^−1^ ZnSO_4_·7H_2_O; and 36 mg·L^−1^ Na_2_MoO_4_·2H_2_O. The pH of FSM and EDTA-TES was adjusted to 7.0 and 6.5, respectively, using 1 M NaOH prior to autoclaving.

MSR-1 cultures were grown in 100 mL DURAN bottles, each with a working volume of 90 mL, under aerobic and microaerobic conditions in an Incu-Shake MAXI (SciQuip Ltd, Newtown, UK) orbital shaker incubator set at 150 rpm and 30°C. Microaerobic conditions promote the formation of magnetosome-producing cells, whereas aerobic conditions inhibit magnetosome production, resulting in non-magnetosome-producing cells. To generate microaerobic conditions, bottles were purged with N_2_ for 30 min to remove all the dissolved O_2_ and sealed with bromobutyl rubber stoppers and later injected with the necessary sterile air volume to achieve ~ 1% O_2_. Aerobic bottles were left with their lids slightly loosened to allow free air exchange. Each bottle was inoculated at a 1:10 ratio and incubated for 60 h, with samples taken every 6–12 h for analysis. During the early exponential growth phase (~ OD_565_ = 0.15), a pulse of iron citrate (final iron concentration of 400 µM) or H_2_O_2_ (final concentration of 150 µM) was added to both aerobic and microaerobic cultures to induce oxidative stress. Experiments were carried out in triplicate. This sampling and stress-induction strategy was chosen to capture dynamic cellular responses during exponential growth while avoiding the lag or stationary phases, which might complicate interpretation. Applying stress at the early exponential phase ensures microaerobic cells already contain magnetosomes, whereas aerobic cells have ceased magnetosome production, allowing a meaningful comparison of oxidative stress effects in their presence or absence.

### Bacterial growth and magnetic cellular response

Bacterial growth was assessed by measuring the optical density of cultures using an Evolution 300 UV-Vis spectrophotometer (Thermo Fisher Scientific, Hemel Hempstead, Herts, UK) at a wavelength of 565 nm (OD_565_). The cellular magnetic response (C_mag_) was evaluated according to a previously described method immediately after the OD_565_ measurements (18). In brief, the spectrophotometer has two pairs of Helmholtz coils mounted on the cuvette holder, one pair oriented perpendicular to the light beam and the other parallel. For magnetic cells, the different alignments result in varying optical densities due to cell migration as a result of the applied magnetic field, whereas non-magnetic cells show no change when the magnetic field orientation is altered, thus maintaining constant optical density. C_mag_ values are then determined by the ratio of OD_565_ measurements for cells aligned parallel and perpendicular to the light beam. C_mag_ values range between 1 and 3, with values exceeding one, indicating the presence of magnetic cells.

### Flow cytometry

Bacterial samples were collected from the liquid cultures, diluted in phosphate-buffered saline solution (PBS), and directly analyzed in a BD Accuri C6 flow cytometer (Becton, Dickinson and Company, Oxford, UK). Flow cytometry was employed to determine relative cell size (FSC-A), cell granularity/complexity (SSC-A), intracellular soluble iron concentration, cell viability, PHA formation, and ROS accumulation. The soluble intracellular iron pool was detected by incubating the cells with Phen Green SK fluorophore (PG-SK) (5 µM) for 10 min; PHA granules were stained with Pyrromethene-546 (Pyr-546) (0.5 μg/mL); cell viability was assessed by incubating the cells with propidium iodide (PI) (100 ng/mL) and bis (1.3-dibutybarbituric acid) trimethine oxonol (BOX) (100 ng/mL) for 2 min; and the presence of ROS was detected by incubating the cells for 30 min at 30°C with CellROX Deep Red (CRDR) fluorophore (5 µM). Cells stained with green (PG-SK, Pyr-546, BOX) and red fluorophores (PI) were excited with a 488 nm solid-state laser, with fluorescence detected using a 533/30 BP filter and a 670 LP filter, respectively. Far-red fluorescent labeled cells (CRDR) were excited with a 640 nm solid-state laser and detected through a 675/25 BP filter.

### Determination of iron content

Inductively coupled plasma optical emission spectroscopy (Thermo Scientific iCAP 7000) coupled with a Teledyne CETAC ASX-520 Random Access Autosampler was employed as an offline analysis to monitor the changes in the intracellular and extracellular iron concentrations of MSR-1 cultures. One mL of each sample was centrifuged to separate cells from the culture media. Supernatants were acidified by adding 10 μL of nitric acid solution (70% vol/vol), whereas cell pellets were digested using 500 μL of the nitric acid solution and incubated at 98°C for 2 h with shaking at 300 rpm. After digestion, the final volume was re-adjusted to 1 mL by adding deionized water. Samples preparation was done and analyzed in triplicate.

### Correlative light and electron microscopy

MSR-1 cells were grown until just before the end of their exponential growth phase. To detect intracellular iron and ROS accumulation, cells were incubated with PG-SK and CRDR, respectively. Vitrification of the samples was performed at the Electron Bio-Imaging Center (eBIC) located at Diamond Light Source, UK’s synchrotron. Approximately 4 µL of the cell suspension was applied to the front of Quantifoil R2/2 grids, with 0.5 µL applied to the back. The grids were then blotted for 6 s using an EM GP2 Automatic Plunge Freezer (Leica Microsystems) before plunge freezing.

After sample vitrification, the grids were transferred to a Leica EM cryoCLEM microscope (Leica Microsystems) to acquire fluorescence data. Regions of interest (ROIs) were identified based on fluorescence signals indicative of intracellular iron and ROS accumulation. The identified ROIs were subsequently imaged using a Titan Krios microscope (Thermo Fisher Scientific), operated at 300 kV with a Gatan K3 detector. Two-dimensional (2D) search maps were acquired at a magnification of 4,800×. For tomographic data collection, a magnification of 8,700× was used, corresponding to a pixel size of 10.51 Å. The total electron dose was 27.53e⁻/Å^²^. Tilt series were acquired from +60° to −60° in 3° increments, with a defocus value of −25 µm. Tomograms were reconstructed using the IMOD software package (University of Colorado, Boulder, CO). Microscopy Image Browser and ChimeraX were employed for volume segmentation and visualization of tomograms (19, 20).

From 2D search maps, several parameters were measured using Fiji (ImageJ) software, including cell length (*n* = 50 cells per condition), number of magnetosomes per cell (*n* = 50 cells per condition), percentage of area occupied by PHA granules relative to the total cell surface area (*n* = 20 cells per condition), and magnetite crystal size (*n* = 500 crystals per condition).

## RESULTS AND DISCUSSION

### MSR-1 physiological responses to stress conditions

To evaluate the physiological responses of MSR-1 to externally induced oxidative stress, the cells were exposed to H_2_O_2_ and iron citrate mid-growth. The physiological impact of these stressors was assessed through measurements of various parameters, such as cellular growth, cell viability, cell morphology and size, PHA formation, and ROS accumulation.

#### MSR-1 growth and viability

MSR-1 was grown under microaerobic and aerobic conditions to obtain magnetosome-producing cells and non-magnetosome-producing cells, respectively; these terms are used consistently throughout the manuscript. When the bacterial cells reached the early exponential growth phase (OD_565_ ≃ 0.15), a pulse of iron citrate (400 µM) or H_2_O_2_ (150 µM) was introduced to induce oxidative stress. The concentrations of iron citrate and H_2_O_2_ used in this experiment were selected based on literature data (11, 13, 21–23) and H_2_O_2_ tolerance tests performed on MSR-1 (Fig. S1).

**Fig 1:**
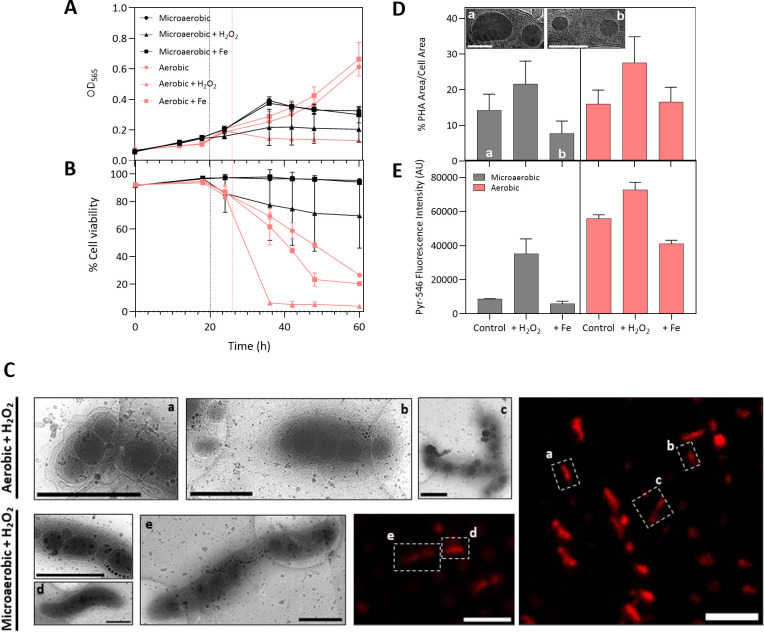
Effects of iron citrate and H_₂_O_₂_ addition on the physiology of MSR-1 cells grown under microaerobic and aerobic conditions. (**A**) Time course of cell growth. (**B**) Cell viability over time, determined by FCM. Vertical lines in graphs A and B indicate the addition of iron and H_₂_O_₂_ pulses in microaerobic (black) and aerobic (red) cultures. (**C**) CryoEM images of MSR-1 cells grown under aerobic and microaerobic conditions after the addition of H_₂_O_₂_ (~150 μM). The cryo-fluorescence image shows cells exposed to H₂O₂, incubated with the CellROX Deep Red fluorophore, which detects the presence of reactive oxygen species (ROS). The areas labeled (**A-E**) in the cryo-fluorescence image correspond to the cells highlighted in the cryoEM images with the same labels. Scale bar = 1 μm (cryoEM) and 10 μm (cryo-fluorescence). (**D**) Percentage of PHA area relative to the total cell area, measured from cryoEM images of MSR-1 cells collected at t = 36 h during the exponential growth phase (*n* = 20 cells/condition). Statistical analysis for this panel was performed using one-way ANOVA, followed by Tukey’s post-hoc test; full results are provided in Table S2. Inset cryoEM images: (a) microaerobic condition and (b) microaerobic condition with iron addition. Scale bar = 300 nm. (**E**) Analysis of PHA content by FCM using Pyr-546 fluorophore at t = 36 h during the exponential growth phase; 25,000 events were analyzed per sample by FCM. Error bars represent the standard deviation of triplicates. FCM = Flow Cytometry; CryoEM = cryo-electron microscopy; Pyr-546 = Pyrromethene-546.

Bacterial growth and cell viability were monitored over a period of 60 h. In microaerobic cultures, the addition of iron citrate did not impact bacterial growth (Fig. 1A) or cell viability (Fig. 1B). The extracellular iron concentration increased from 6 mg/L to approximately 20 mg/L following the iron citrate pulse (Fig. S2). Although previous work suggests that a 20 mg/L concentration should be toxic for MSR-1 (22, 23), our findings reported in another published study (24), and again herein, indicate that neither bacterial growth nor cell viability was affected by the increase in the extracellular iron concentration (across the studied concentration range up to 55 mg/L; Fig. S3A). In contrast, although bacterial growth in aerobic cultures remained unaffected by the addition of iron citrate (Fig. 1A), cell viability declined more rapidly compared with control conditions (Fig. 1B). This accelerated decline is tentatively attributed to potential cellular damage caused by the Fenton reaction (10).

On the other hand, the addition of H_2_O_2_ significantly impacted bacterial growth (Fig. 1A). Aerobic growth was completely ceased as the OD_565_ values dropped from 0.19 to 0.13, likely due to cell lysis. Meanwhile, the growth rate under microaerobic conditions was reduced but not completely restrained (OD_565_ = 0.22), relative to the control (OD_565_ = 0.37). Aerobic cultures also exhibited a much steeper decline in cell viability (Fig. 1B), with viability dropping from 85% to less than 10% immediately after the H_2_O_2_ pulse, whereas microaerobic cultures only decreased by 25% relative to the control. These effects can be attributed to the interaction of H_2_O_2_ with the bacterial cell membrane, which can cause lipid peroxidation and disrupt membrane integrity (25, 26). Oxidative stress can also reduce proton motive force by inhibiting transport across the cell membrane, which reduces ATP production and consequently inhibits cell growth, leading to cell death (27).

There are only a few studies published on the impact of H_2_O_2_ on MTB growth. The majority of which were performed under microaerobic conditions solely and started to see a significant impact on bacterial growth at 200–300 µM concentrations in the media (11, 13, 21). These studies typically exposed bacteria to different H_2_O_2_ concentrations from the start of the culture, whereas our study introduces a pulse of H_2_O_2_ during mid-growth, which may explain the differences in the observed behavior. By introducing H_₂_O_₂_ as a pulse during mid-growth, this study specifically focuses on the physiological responses of MSR-1 to sudden oxidative perturbations, providing insight into how cells cope with acute stress rather than long-term adaptation. Under these conditions, microaerobic cells tolerated the iron pulse without affecting growth or viability and were lightly impacted by H_₂_O_₂_, whereas aerobic cells showed decreased viability with iron and were severely affected by H_₂_O_₂_ exposure.

#### MSR-1 cell size and morphology

The impact of external oxidative stress on MSR-1 cell morphology and size was analyzed, with a focus on the changes induced by doping high iron and H_2_O_2_ concentrations. FCM analysis provides insights into cell size and granularity, whereas cryo-electron microscopy (cryoEM) images offer high-resolution details of cellular structures. Together, these methodologies reveal the extent to which oxidative stress affects MSR-1 cells, highlighting differences in their structural and intracellular organization. Samples for most analyses were collected every 6–12 h to monitor temporal dynamics; however, cryoEM was performed during the exponential growth phase (only at t = 36 h) to capture the full effects of the stress factors before the stationary phase or cell death.

The obtained results indicate that the addition of iron does not result in any noticeable morphological differences when grown under microaerobic or aerobic conditions. In contrast, as shown in Fig. 1C, H_2_O_2_ doping induces various effects on MSR-1 morphology. Aerobic cells subjected to H_2_O_2_ exhibited significantly more structural cell damage compared with microaerobic cells. The cell membrane of aerobic cells was notably compromised, with many cells losing their characteristic spirillum shape and becoming smaller and more rounded (Video S1). Some cells also appeared to be broken. Although some damage was observed in microaerobic cells exposed to H_2_O_2_, it was considerably less severe compared with the damage seen in aerobic conditions. This difference in structural cellular damage correlates with the larger decrease in cell viability observed in Fig. 1B under H_2_O_2_-doped aerobic conditions, where viability was reduced by 80%, compared with a 25% reduction in H_2_O_2_-doped microaerobic cultures. The sensitivity of bacterial cells to H_2_O_2_ has been documented in other bacterial species. For instance, *Campylobacter jejuni* changes from a spiral-shaped to a coccoid form upon interaction with ROS (28), whereas *Escherichia coli* turns from a bacillus form to a coccoid form (29). These morphological changes occur as bacteria attempt to repair the membrane damage caused by ROS, which, if unrepaired, may lead to DNA damage and cell death (30). Table 1 displays the differences in cell length under iron and H_2_O_2_ doping conditions, with cell length measured from cryoEM 2D search maps using Fiji software. The results revealed that exposure to H_2_O_2_ led to significantly shorter cells, about half the size of those in the control group, whereas iron exposure had no effect on cell size. This reduction in size is likely due to morphological changes caused by lipid peroxidation of the cell membrane from H_2_O_2_ exposure. Our findings also indicate that aerobic cells were significantly longer (8.3 ± 3.1 µm) than microaerobic cells (6.1 ± 1.8 µm) (one-way ANOVA with Tukey test, *P* < 0.01; Table S1). Some aerobic cells even reached double the size of some microaerobic cells, measuring up to 16 µm, which has also been previously observed by others (9). This difference in cell length can be clearly observed in Fig. S4, which shows representative aerobic and microaerobic cells. The analysis of the forward scatter (FSC-A) data from the FCM, which provides information regarding cell size, is in line with the microscopy results, showing that aerobic cells generally exhibited longer dimensions than microaerobic cells (Fig. S5A). In many bacteria, cell size is influenced by growth rate, but this relationship has not, to our knowledge, been systematically studied in MTB. Thus, although differences in oxygen and culture conditions may influence growth rate and thereby cell size, we cannot confirm that the same growth‐rate/size rules apply to MSR-1. Besides oxygen availability, other changes in the growth conditions or other stress factors, such as variations in lactic acid and nitrate concentrations in the media (18), the method of cultivation (plates *vs*. liquid cultures) (31), or exposure to UV-B radiation (32), have been shown to affect the size and morphology of MTB. Monitoring and controlling cell size is therefore important, as this phenotypic feature can significantly impact bioprocess efficiency, influencing both upstream factors like nutrient uptake and downstream processes such as cell disruption and product recovery (33).

**TABLE 1:** Effects of doping iron citrate and H_₂_O_₂_ on cell length (*n*=50/condition), magnetosome content (*n* = 50/condition), and magnetite crystal size (*n* = 500/condition) in microaerobic and aerobic MSR-1 cells^*a*^

	Cell length (µm)		Magnetosomes/cell		Magnetite size (nm)	
	Microaerobic	Aerobic	Microaerobic	Aerobic	Microaerobic	Aerobic
Control	6.1 ± 1.8	8.3 ± 3.1	24.6 ± 5.5	11.8 ± 7.9	48.6 ± 10.5	34.5 ± 9.4
H_₂_O_₂_ addition	3.7 ± 1.8	4.5 ± 1.8	20.7 ± 7.2	8.0 ± 4.7	43.4 ± 10.6	43.1 ± 10.7
Iron addition	5.9 ± 1.0	7.5 ± 2.1	31.8 ± 7.0	8.4 ± 5.0	48.2 ± 11.4	35.0 ± 8.1

^*a*^ All measurements were obtained from cryoEM 2D search maps using Fiji software. Statistical analysis details (one-way ANOVA followed by Tukey’s post-hoc test) are provided in Tables S1, S3, and S4.

#### PHA granule accumulation

FCM was also used to assess intracellular complexity or granularity through side scatter (SSC-A) measurements. In MSR-1, aside from magnetosome chains, cells typically accumulate intracellular inclusions such as phosphate or PHA granules (34).

As is well known, PHA synthesis is notably enhanced under conditions of nutrient deficiency or oxidative stress, particularly when there is an excess of carbon source (35). Monitoring PHA synthesis is important because PHAs serve as reservoirs for reducing power, thereby helping us to manage intracellular redox balance and mitigate ROS formation (35), and their accumulation can function as an indirect indicator of cellular stress. By sequestering reducing equivalents (e.g., NADH and NADPH), PHA synthesis prevents excess reducing agents from fueling uncontrolled ROS production and oxidative damage (36). During periods of starvation, PHAs can be degraded to release monomers that enter central metabolic pathways (e.g., the TCA cycle and glycolysis), thereby restoring reducing power necessary for maintaining redox balance (37).

Here, PHA granules were stained with the Pyr-546 fluorophore and monitored by FCM. Changes in cell granularity (SSC-A) and Pyr-546 intensity values over time are shown in Fig. S5B and C**,** respectively. Both plots clearly indicate that aerobic conditions exhibited higher fluorescence and SSC-A values, suggesting increased PHA granule synthesis under these conditions. Our results are in accordance with the investigation of Su *et al*. using the AMB-1 strain in which they observed a decrease in PHA content under anaerobic conditions and an increase under aerobic conditions (38).

Figure 1D and E display the percentage of PHA inclusions in the cytoplasmic space and Pyr-546 fluorescence intensity values, respectively, for MSR-1 cells during the exponential growth phase, when samples were collected for cryoEM imaging. Both analytical approaches (FCM and cryo-EM) showed similar trends in PHA content across the different conditions. Under aerobic conditions, iron addition did not result in significant differences in PHA content as cells exhibited similar values to the control conditions (16% PHA area/cell area). However, under microaerobic conditions, the PHA area relative to total cell area decreased significantly from 14% to 8% with iron addition (one-way ANOVA with Tukey test, *P* < 0.01; Table S2). Inset cryo-EM images in Fig. 1D further show that the size of PHA granules was significantly reduced after iron addition under microaerobic conditions. Interestingly, in other bacterial species, such as *Methylobacterium extorquens*, a byproduct of PHA degradation (methyl-esterified 3-hydroxybutyrate) has been shown to exhibit powerful antioxidative activity against hydroxyl radicals (39). Although this mechanism has not been demonstrated in MSR-1, it highlights the possibility that PHA’s metabolism may contribute more broadly to oxidative stress mitigation in MTB. In other bacterial species, such as *Cupriavidus necator*, the synthesis of PHA has been shown to increase under moderately elevated levels of oxidative stress (≤10 mM H₂O₂) (40, 41), supporting the notion that PHA accumulation can be part of a general stress response. In our study, the higher PHA content observed in H_2_O_2_-exposed cells (22% and 28% for microaerobic and aerobic cultures, respectively) may reflect a similar cellular response to oxidative stress or, alternatively, be biased by the changes in cell size and morphology resulting from H_2_O_2_-induced cell damage. For instance, broken cells had cytoplasmic leakage, reducing the total cell area and thereby increasing the ratio of PHA to cytoplasmic space, due to the cell damage rather than increased PHA production.

Monitoring PHA formation is important in bioprocessing since its accumulation reflects cellular stress and signals changes in culture physiology. Excessive PHA can divert carbon and reducing power away from magnetosome biosynthesis (18), lowering yields, whereas residual granules complicate downstream purification and reduce final product quality (42). Incorporating PHA monitoring into process control strategies can therefore enhance both culture performance and magnetosome recovery. This study provides one of the few assessments of PHA dynamics in MTB, revealing condition-dependent variations linked to environmental stress factors. These insights expand our understanding of PHA physiology in MTB and underscore the need for further studies to elucidate the environmental and metabolic triggers that regulate their formation.

#### Intracellular ROS accumulation

MSR-1 cells were stained with CellROX Deep Red (CRDR) fluorophore and analyzed by FCM to investigate the accumulation of ROS. This fluorescent probe has been effectively employed for ROS detection in other gram-negative bacteria such as *Escherichia coli* (43) and *Fusobacterium nucleatum* (44).

**Fig 2:**
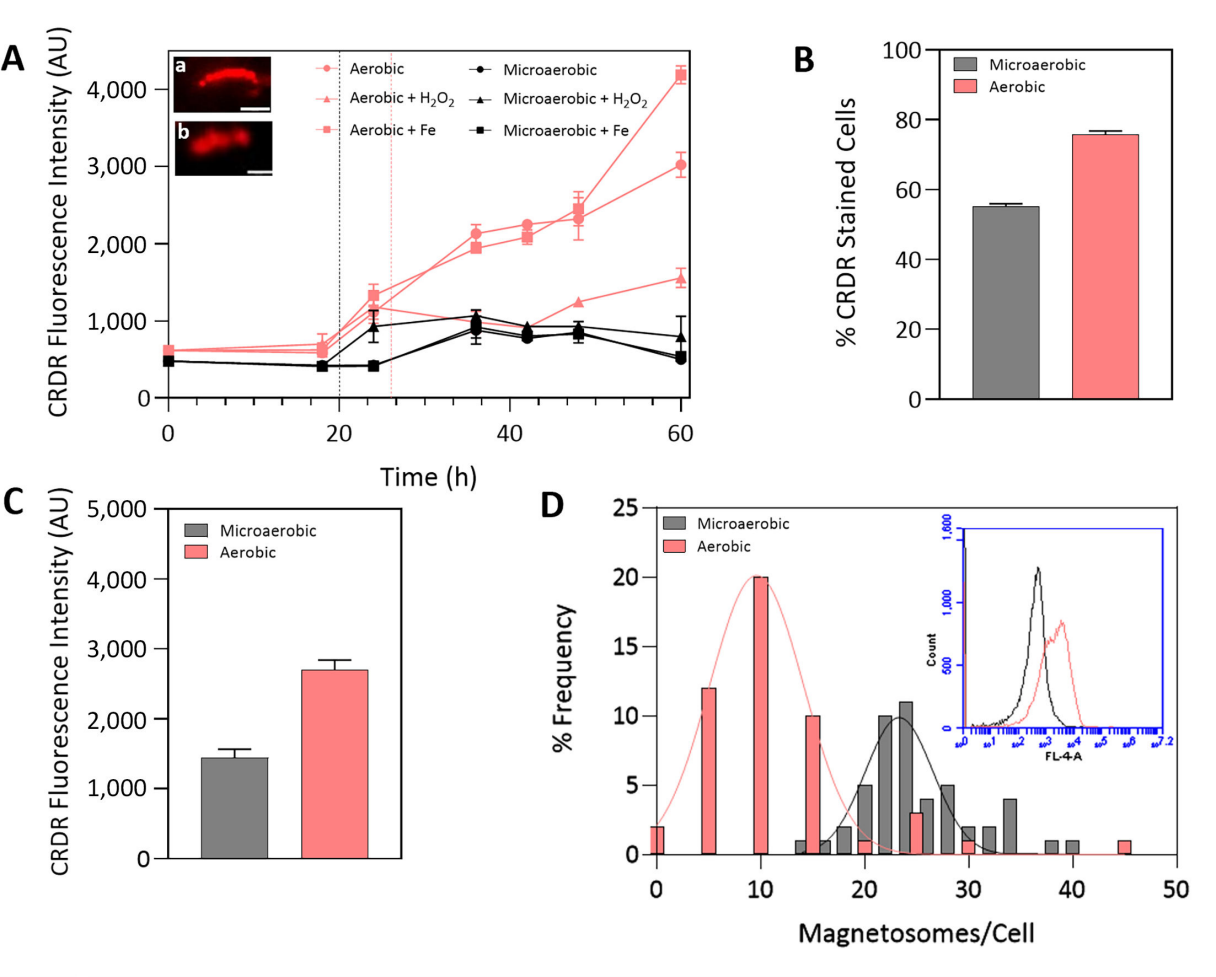
(**A**) Accumulation of ROS in CRDR-stained MSR-1 cells by FCM. Inset cryo-fluorescence images: (a) aerobic control condition and (b) aerobic condition with H_₂_O_₂_ addition. Scale bar = 2 μm. Comparison between aerobic and microaerobic MSR-1 cells of (**B**) the percentage of CRDR-stained cells and (**C**) the fluorescence intensity values of the CRDR-stained cells during the exponential growth phase (t = 36 h). (**D**) Magnetosome chain length histogram distribution of aerobic and microaerobic cells. The inset graph corresponds to CRDR fluorescence histograms obtained from FCM analysis of aerobic and microaerobic cells. Vertical lines in graph (**A**) indicate the addition of iron and H_₂_O_₂_ pulses in microaerobic (black) and aerobic (red) cultures; 25,000 events were analyzed per sample by FCM. Error bars represent the standard deviation of triplicates. CRDR = CellROX Deep Red; FCM = Flow cytometry; AU = arbitrary units.

Figure 2A illustrates ROS accumulation of MSR-1 cells exposed to oxidative stress conditions over time. Generally, cells under aerobic conditions exhibited significantly higher levels of intracellular ROS compared with those under microaerobic conditions. In microaerobic cultures, the addition of iron did not lead to an increase in ROS production. This was consistent with complementary experiments in which MSR-1 cells were grown with varying iron concentrations (up to 1 mM), as shown in Fig. S3B. In previous work, gene expression analysis performed during cell growth and magnetosome synthesis revealed that genes associated with ferric reductases, ferrous transport system, and ROS scavenging are highly expressed when exposed to high iron concentrations (45). The upregulation of ROS scavenging-related genes under high iron concentrations presumably facilitates the ability of MTB to uptake large amounts of iron and thrive in iron-rich environments. However, under aerobic conditions, ROS production spiked 22 h after cells were exposed to an iron pulse, most likely as a result of the Fenton reaction.

The addition of H_2_O_2_ resulted in a moderate increase in ROS accumulation for microaerobic conditions compared with the control (Fig. 2A and 1C). Surprisingly, H_2_O_2_-aerobic cultures exhibited lower CRDR fluorescence values compared with the control conditions when analyzed by FCM. However, fluorescence microscopy images (insets in Fig. 2A) revealed similar fluorescence levels to those achieved in aerobic control conditions. Aerobic cells were significantly damaged under the influence of H_2_O_2_, resulting in smaller, more compact, and even fragmented cells (Fig. 1C). This loss of membrane integrity likely led to partial leakage of the CRDR dye, which can cause an apparent decrease in fluorescence intensity detected by FCM (46). Nevertheless, microscopy still revealed strong fluorescence within the damaged cells, consistent with high intracellular ROS levels. This complementary interpretation underscores the value of combining FCM and fluorescence imaging to better capture cellular responses under stress conditions.

Using the gating capability of FCM, a more in-depth analysis of ROS distribution was conducted by comparing low magnetosome-producing cells (aerobic) with high magnetosome-producing cells (microaerobic), as magnetosomes are believed to decrease and eliminate ROS due to their peroxidase-like activity (14). Analysis of the proportion of CRDR-stained cells (ROS^+^) in Fig. 2B revealed a 20% difference between these two conditions, with 55% of ROS^+^-microaerobic cells compared with 75% ROS^+^-aerobic cells. ROS^+^ cells were defined based on fluorescence above the threshold set using non-stained control samples, indicating the presence of ROS. A closer examination of the mean fluorescence intensity values of ROS^+^ cells (Fig. 2C) reveals that ROS^+^-aerobic cells showed double the fluorescence compared with ROS^+^-microaerobic cells, indicating a clear contrast in ROS levels between the two conditions. Importantly, no correlation was observed between FSC-A and CRDR fluorescence intensity (Fig. S6), confirming that the observed differences in fluorescence are not attributable to cell size but rather reflect physiological differences in ROS accumulation between conditions.

Based on FSC-A and SSC-A plots, two distinct populations can be identified for both microaerobic and aerobic conditions (Fig. S7A). The first population (P1) consists of smaller cells with fewer granules, constituting approximately 70% of the total cell population, whereas the second population (P2) consists of larger cells with higher granule content, representing the remaining 30%. Both aerobic populations also exhibited a much higher percentage of ROS^+^ cells (P1: 70%; P2: 90%) compared with microaerobic populations (P1: 45%; P2: 70%) (Fig. S7B). A similar trend to the one previously observed is clear, with aerobic populations displaying at least twice the level of fluorescence compared with microaerobic populations (Fig. S7C). It is also noteworthy to mention that the difference in fluorescence values between P1 and P2 under microaerobic conditions is only 1.6-fold, whereas under aerobic conditions, it is 2.2-fold. This indicates that magnetosome-producing cells (microaerobic) display both lower fluorescence heterogeneity and lower ROS levels compared with non-magnetosome-producing cells (aerobic). This fluorescence heterogeneity is also reflected in the FCM fluorescence histograms (Fig. 2D inset). The CRDR fluorescence peak observed in the CRDR histograms for microaerobic cells was narrower compared with the broader distribution observed for aerobic cells. This pattern was in line with the magnetosome chain length distribution plots for both conditions (Fig. 2D), where the distribution of magnetosome chain lengths in aerobic cells was much more dispersed than in microaerobic cells. The aerobic distribution has a coefficient of variation (CV) of 135%, indicating higher relative dispersion compared with the microaerobic distribution, which has a CV of 96%. This suggests that the higher CRDR fluorescence heterogeneity observed in aerobic conditions is potentially correlated with greater heterogeneity in magnetosome content. Considering that prior studies have demonstrated that MSR-1 mutants deficient in magnetosome production exhibit significantly higher ROS levels than the wild-type strain capable of producing magnetosomes, even when both were grown under microaerobic conditions (14), our findings are consistent with a potential correlation between the heterogeneity in magnetosome content and ROS levels, as reflected by the observed fluorescence heterogeneity. Although the functional implications of this fluorescence heterogeneity remain unclear, its consistent association with both ROS levels and magnetosome content in our observations and in previous reports suggests that it reflects underlying physiological regulation in MSR-1 and should be the focus of future investigations.

### Impact of stress conditions on magnetosome biomineralization

The regulation of iron homeostasis is intrinsically linked with the bacterial response to oxidative stress, reflecting a coordinated control system to manage these demands (47). In MTB, this coordination is particularly crucial, as iron is a key component in magnetosome biomineralization. In here, the impact of stress conditions on magnetosome formation in magnetosome-producing and non-magnetosome-producing MSR-1 cells is further explored. Understanding these interactions and regulatory mechanisms is vital for elucidating how MTB manage iron homeostasis while minimizing oxidative damage. Thus, it is of special interest to improve current bioprocessing strategies.

#### Magnetosome chain length, morphology, and crystal size

Changes in the culture media and external environmental factors, such as temperature, pH, dissolved oxygen levels, and iron concentrations can affect the number and shape of the magnetosome crystals (4, 48–50). In this study, the impact of adding H_₂_O_₂_ and iron citrate on magnetosome content, chain morphology, and crystal size was analyzed using cryoEM (Table 1).

Despite the suppression of magnetosome biomineralization under aerobic conditions (9), magnetosome chains were still present due to the high magnetism of the inoculum used (C_mag_ = 2.7, Video S2). However, as displayed in Table 1, the chains in aerobic cells were only half as long (11.8 ± 7.9 magnetosomes/cell) as those in microaerobic cells (24.6 ± 5.5 magnetosomes/cell) (Video S3). The addition of iron and H_2_O_2_ to aerobic cells did not significantly alter the number of magnetosomes per cell. In contrast, under microaerobic conditions, the iron pulse resulted in the formation of significantly (one-way ANOVA with Tukey test, *P* < 0.01; Table S3) longer magnetosome chains (31.8 ± 2.1 magnetosomes/cell). These findings align with our previously published study, which demonstrated that increasing extracellular iron content promotes the formation of longer magnetosome chains (24).

**Fig 3:**
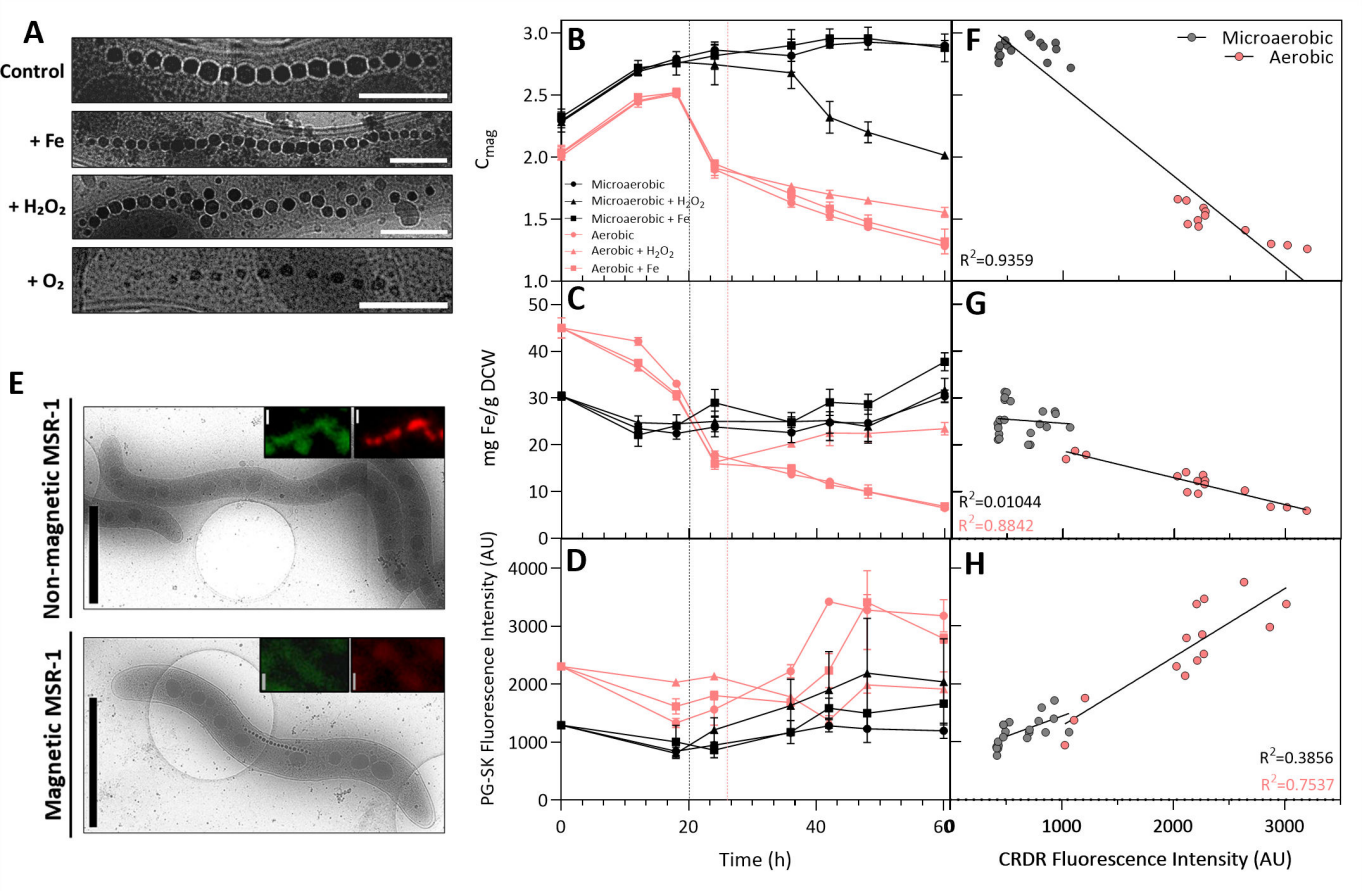
Analysis of iron dynamics and ROS accumulation during magnetosome biomineralization. (**A**) Effects of the addition of iron citrate (+ Fe), hydrogen peroxide (+ H₂O₂), or aerobic conditions (+ O_2_) on the magnetosome chain structure. Scale bar = 300 nm. (**B**) C_mag_, (**C**) total intracellular iron content, and (**D**) labile ferrous iron levels in MSR-1 cells grown under various stress conditions over a period of 60 h. Vertical lines in (**B-D**) indicate the addition of iron and H₂O₂ pulses in microaerobic (black) and aerobic (red) cultures. (**E**) Comparison between non-magnetic (aerobic) and magnetic (microaerobic) MSR-1 cells using PG-SK and CRDR fluorescence, alongside their corresponding cryoEM images. Scale bar = 2 μm. (**F-H**) Correlation of C_ma_g (*n*=30), total intracellular iron content (*n*=36), and labile ferrous iron levels (*n*=35) with intracellular ROS accumulation of microaerobic and aerobic cells, respectively. Each data point in (F–H) corresponds to the same measurements shown in (B–D), but only for the control aerobic and microaerobic conditions. Labile ferrous iron levels and ROS accumulation were measured using (**D-H**) flow cytometry (FCM) or (**E**) cryo-fluorescence microscopy with PG-SK (green fluorescence) and CRDR (red fluorescence) fluorophores, respectively; 25,000 events were analyzed per sample by FCM. Error bars represent the standard deviation of triplicates. CRDR = CellROX Deep Red; PG-SK = PhenGreen SK; AU = arbitrary units.

As shown in Fig. 3A, the spacing between magnetite crystals in aerobic cells was larger compared with microaerobic cells (9). Although the addition of iron did not induce any noticeable changes in magnetosome chain morphology, the exposure to H_2_O_2_ affected the chain structure. The oxidative stress induced by H_2_O_2_ led to the loss or deformation of the typical needle-like shape and, in some cases, caused chain fragmentation. MamK is an actin-like protein responsible for organizing the magnetosome chain along the cell axis (51). Similar to what has been observed in eukaryotic cells, where H_2_O_2_ disrupts actin dynamics [25], oxidative stress from H_2_O_2_ could have impaired the function of MamK, leading to the observed structural alterations.

Measurement of magnetite crystal size under the different culture conditions (Table 1) from 2D cryoEM search maps revealed that microaerobic magnetosomes were significantly larger (48.6 ± 10.5 nm) than those present in aerobic cells (34.5 ± 9.4 nm) (one-way ANOVA with Tukey test, *P* < 0.01; Table S4) (52). Iron addition did not enlarge magnetosome crystals under microaerobic conditions, but H_2_O_2_ exposure resulted in significantly smaller crystals. This reduction in magnetite crystal size caused by H_2_O_2_ is consistent with previous studies in which MSR-1 cells were exposed to similar H_2_O_2_ concentrations (200 µM) (53). Although other research has reported a correlation between increased extracellular iron concentration and larger magnetite crystals in the AMB-1 strain (54), our results did not show an increase in magnetite size upon iron addition (48.2 ± 11.4 nm). This discrepancy may be due to differences in experimental protocols, as bacteria were grown at different iron concentrations from the start, whereas in this study, the iron pulse was added mid-growth, potentially affecting magnetite growth differently. Other studies have reported that increasing extracellular iron concentration can alter the morphology of magnetosome crystals rather than their size when iron uptake rates are affected (48). Specifically, magnetosomes grown under high iron uptake rates displayed cuboidal morphologies, whereas those grown with slower iron uptake rates exhibited the classical cubo-octahedral morphology. These observations highlight the complexity of magnetosome biomineralization and emphasize the critical role of environmental factors in shaping magnetosome morphology. Beyond these individual effects, our findings underline that the physiological and phenotypic responses involved are interconnected and cannot be fully understood in isolation. By combining complementary approaches, this study provides a holistic view of magnetosome formation as a multifactorial process governed by both environmental and intracellular parameters, advancing our understanding of biomineralization within its broader physiological context rather than through isolated variables.

#### Interplay between iron content and ROS accumulation

Figure 3B shows C_mag_ values recorded at regular intervals to enable in-line analysis of magnetosome content over time. The determination of the number of magnetosomes per cell by cryoEM was conducted at a single time point (during the exponential growth phase, t = 36 h); thus, the collection of C_mag_ values ensured a more continuous assessment of magnetosome content, complementing the cryoEM data. As aforementioned, the inoculum used was highly magnetic; hence, C_mag_ values for microaerobic conditions were consistently high from the beginning and maintained throughout the experiment (C_mag_ = 2.3–2.9). For aerobic cultures, there was a period of adaptation of approximately 20 h in which cells switched to their aerobic metabolism. Afterward, C_mag_ values began to decrease, down to 1.3, indicating the suppression of magnetosome production (9, 18). There were practically no C_mag_ differences between the control and the iron-doping conditions for both microaerobic and aerobic conditions. Notably, differences in C_mag_ values were only observed in conditions where H_2_O_2_ was added (Fig. 3B). These observations showed some inconsistencies with the cryoEM magnetosome content results displayed in Table 1, where no significant differences in magnetosome content were found upon H_2_O_2_ addition. Although cryoEM provides precise, high-resolution information on cell morphology and the exact number of magnetosomes per cell, it is an offline, low-throughput technique. In contrast, C_mag_ offers a rapid, in-line, and qualitative assessment of culture’s magnetism, although its values can be influenced by factors such as cell length and morphology (31). The discrepancies observed under H_₂_O_₂_ stress are therefore likely explained by morphology-dependent effects on C_mag_, whereas cryoEM more accurately reflected the actual number of magnetosomes per cell at the measured time point. Together, the two methods provide complementary perspectives on the impact of stress on MSR-1 cells.

Total intracellular iron content results (Fig. 3C) followed a trend similar to that observed in the C_mag_ results. In aerobic cultures, the iron concentration decreased up to 7 mg/g DCW, whereas in microaerobic cultures, the iron concentration remained consistently high, around 30 mg/g DCW over time (9, 52). Unlike C_mag_ data, microaerobic conditions with added iron displayed higher intracellular iron concentrations (38 mg/g DCW), likely due to the presence of longer magnetosome chains (Table 1). After H_2_O_2_ addition in aerobic conditions, the intracellular iron levels remained constant. This was attributed to the cease of cell growth and significant cell viability reduction (Fig. 1) that these cells suffered under these conditions.

Measurement of the total intracellular iron content has been commonly used to assess magnetosome content. However, as noted in previous studies, there are different intracellular iron pools distinct from magnetite (24, 55, 56). In addition to its role in magnetite biomineralization, iron is also required for other general iron-dependent biochemical reactions (57, 58) or stored in proteins such as ferritins to prevent toxicity (59). Consequently, some studies have suggested that only a fraction of the total intracellular iron (25%–45%) corresponds to magnetite (55, 56). Hence, the importance of also tracking changes of the soluble Fe^2+^ intracellular iron pool. This is particularly important because free iron (Fe^2+^) can react with H_2_O_2_ to generate hydroxyl radicals.

Labile Fe^2+^ intracellular iron concentration was monitored by FCM using PG-SK fluorophore, which quenches upon binding to free Fe^2+^ ions (31, 60). As shown in Fig. 3D, aerobic cultures displayed higher green fluorescence values compared with microaerobic cultures, indicating a notably lower presence of Fe^2+^ under aerobic conditions. This observation was corroborated by cryo-fluorescence microscopy, as shown in Fig. 3E. Under microaerobic conditions, the addition of H_2_O_2_ led to a decrease in Fe^2+^ levels. This decrease was likely due to oxidation of Fe^2+^ to Fe^3+^ via the Fenton reaction, as ROS levels also increased (Fig. 2A).

**Fig 4:**
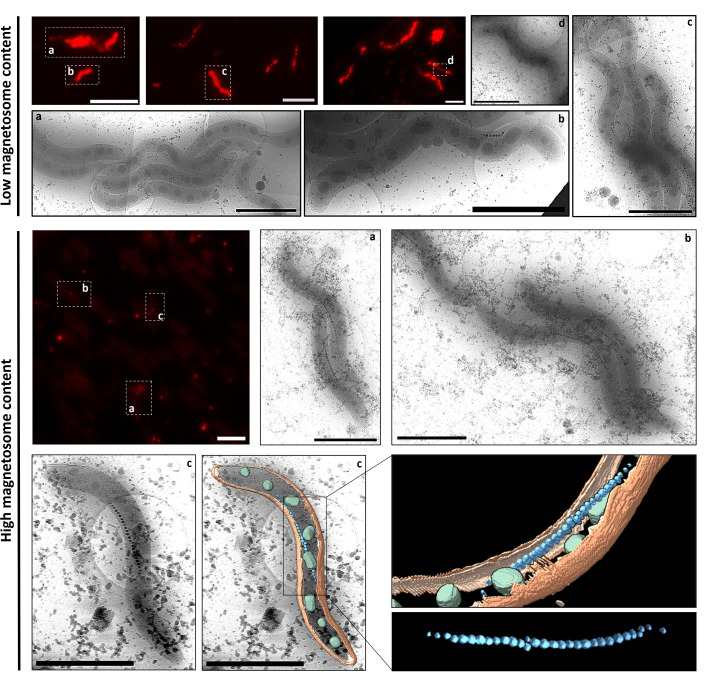
Comparison of intracellular ROS accumulation of MSR-1 cells containing high or low magnetosome content. Cells were stained with CellROX Deep Red fluorophore to detect ROS presence. CryoEM images of selected cells are matched to the corresponding cryo-fluorescence images as indicated by the same letter labels. A representative 3D volumetric representation of a highly magnetic cell is included. Magnetosome chain is colored in blue, PHA granules in green, outer membrane in orange, and inner membrane in light orange (Video S4). Scale bar = 2 μm (cryoEM) and 10 μm (cryofluorescence).

In a previous correlative microscopy study (24), we found a direct correlation between the number of magnetosomes and the intracellular Fe^2+^ iron pool, in which cells with longer magnetosome chains exhibited higher Fe²^+^ levels than those with shorter chains. It also showed that not all internalized Fe²^+^ was used for magnetite synthesis, as a substantial labile Fe²^+^ pool remained even after biomineralization was complete (61). Given these observations, in this study, we aimed to elucidate the relationship between ROS accumulation, the intracellular soluble iron pool, and magnetosome content. To this end, C_mag_ values (Fig. 3F), total intracellular iron content (Fig. 3G), and intracellular Fe^2+^ levels (Fig. 3H) were correlated with ROS accumulation. Additionally, the cryoCLEM approach was employed to obtain data at the single-cell level (Fig. 3E and 4) by comparing magnetic (microaerobic) and non-magnetic (aerobic) cells.

Evidence suggests that MSR-1 possesses homologs for the global regulators Fur (62), OxyR (21), and IrrB (63), indicating that these regulators work together to fine-tune iron homeostasis and oxidative stress responses during magnetosome biomineralization. In MSR-1, Fur is involved in regulating the transcription of Fe^2+^ transport systems *feoAB1* and *feoAB2*, as well as genes involved in oxidative stress responses, such as *katG* (catalase) and *sodB* (superoxide dismutase) (62, 64). This highlights Fur’s dual role in managing both iron levels and oxidative stress. OxyR, a key regulator of the oxidative stress response, responds to H_2_O_2_ by upregulating genes that mitigate ROS damage such as *katG* and *dps* (iron storage protein) (65). Therefore, OxyR also indirectly influences iron metabolism by promoting iron sequestration and reducing free iron levels, thus helping in lower ROS formation (11). The combined actions of Fur and OxyR underscore the complex interplay between iron regulation and oxidative stress management.

Our results, shown in Fig. 3F, revealed an inverse correlation between C_mag_ values and ROS accumulation. Specifically, higher C_mag_ values, which indicate greater magnetosome content, were associated with lower ROS levels. This suggests that increased magnetosome formation might be linked to reduced oxidative stress, potentially due to iron sequestration within magnetite or their peroxidase-like activity (14). This trend is further supported by single-cell correlative microscopy images presented in Fig. 4, where MSR-1 cells with higher magnetosome content exhibited lower CRDR fluorescence than cells with lower magnetosome content.

By analyzing the correlation between the total intracellular iron content and ROS levels (Fig. 3G), distinct patterns between microaerobic and aerobic conditions were observed. It was expected that, due to the Fenton reaction, cells with higher intracellular iron concentrations would exhibit elevated ROS levels, as excess iron can catalyze ROS formation. However, under microaerobic conditions, which exhibited high intracellular iron concentrations over time, did not show a significant correlation between total iron content and ROS accumulation, suggesting that MSR-1 cells may regulate intracellular iron in a way that prevents excessive ROS formation under microaerobic conditions. Such regulation could involve iron-storage proteins, such as bacterioferritins, which have been reported in MSR-1 and linked to oxidative stress responses rather than magnetosome biomineralization (59). Their expression is also regulated by the OxyR ROS regulator, further supporting a role in mitigating iron-induced oxidative stress (11). In contrast, in aerobic conditions, a decrease in total intracellular iron content corresponded with an increase in ROS levels. This may result from a reduction in the number of magnetosomes per cell, thereby diminishing the capacity of MSR-1 cells to mitigate oxidative stress through magnetosome-associated ROS scavenging activity (13, 14). Despite Fur acting directly as a global regulator responding to iron levels (66), oxidative stress (such as that caused by high oxygen conditions) can influence iron homeostasis and indirectly affect Fur activity. Cells might respond by more tightly regulating iron uptake and storage to minimize the availability of free iron that could participate in ROS generation. This response could hypothetically involve Fur-mediated pathways to ensure iron levels are kept within a safe range, although Fur’s primary direct response is to iron rather than to ROS or oxygen levels (62).

The correlation between labile Fe²^+^ (detected by PG-SK fluorescence) and ROS levels also varied notably depending on oxygen availability (Fig. 3H). Under microaerobic conditions, no significant correlation was observed. However, under aerobic conditions, a clear inverse correlation was observed: lower Fe²^+^ levels, indicated by higher PG-SK fluorescence, were associated with increased ROS accumulation. This relationship can be explained by the Fenton reaction. Since PG-SK has a high affinity for Fe²^+^ but not for Fe³^+^ (67), its fluorescence increases as Fe²^+^ is oxidized and becomes less detectable by PG-SK, whereas ROS levels rise. Correlative microscopy images in Fig. 3E further illustrate this situation, showing that magnetic cells exhibited low PG-SK and CRDR fluorescence, whereas non-magnetic cells exhibited high fluorescence. The images shown are representative of the cell population, for which the same trends were consistently observed.

Overall, these results suggest that magnetosomes likely play a significant role in mitigating oxidative stress, but this role cannot be completely disentangled from the broader physiological effects of oxygen availability in the absence of magnetosome-deficient mutants. The observed correlations, therefore, reflect the complex interplay between antioxidant mechanisms, iron homeostasis, and magnetosome biomineralization. Even within this multifactorial context, the integrated analysis presented here expands our understanding of magnetosome physiology and emphasizes the importance of simultaneously monitoring these interconnected parameters to enhance both fundamental knowledge and bioprocess efficiency.

### Conclusions

This study highlights the complexity of MSR-1 physiological responses, showing that phenotypic changes, including cell size and morphology, magnetosome formation, ROS and PHA accumulation, cell viability, and intracellular iron levels, are interconnected and influenced by multiple environmental stress factors such as oxygen availability, iron concentration, and oxidative stress. By specifically focusing on responses to sudden oxidative perturbations, this work provides insight into how cells cope with acute stress rather than long-term adaptation. Our findings reveal distinct differences in how microaerobic magnetosome-producing and aerobic non-producing MSR-1 cells respond to sudden environmental changes. Under microaerobic conditions, cells were smaller, accumulated less PHAs, maintained high magnetosome and iron levels, and experienced minimal oxidative stress, whereas aerobic cells were larger, with reduced magnetosome and iron levels, accumulated more PHAs, increased ROS levels, and a significantly decreased viability. In line with previous reports on magnetosome-deficient mutants, our findings indicate that magnetosomes are likely to play an important role in mitigating oxidative stress, whereas oxygen concentration and related metabolic processes may also contribute to shaping ROS levels. This highlights the complex interplay between antioxidant mechanisms, iron regulation, and magnetosome biomineralization. By combining complementary high-resolution and high-throughput techniques (e.g., cryoCLEM, FCM, and ICP-OES) in a single integrated experimental design, this work provides a unique, multi-dimensional view of MTB responses. Beyond fundamental insights into MTB biology, these findings have practical implications for bioprocessing, as monitoring interconnected physiological parameters can improve culture robustness, magnetosome yield, and downstream processing efficiency. Overall, this work establishes a benchmark for future studies on MTB stress responses and underscores the value of integrated, correlative analytical approaches in revealing complex microbial physiology.

## SUPPLEMENTAL MATERIAL

Supplemental material mbio.03265-25-s0001.docx Supplemental figures, tables, and video captions.

Video S1 mbio.03265-25-s0002.avi MSR-1 cryo-electron tomography showing the damaging effects of H2O2 exposition.

Video S2 mbio.03265-25-s0003.avi Representative cryo-electron tomography of MSR-1 cells grown aerobically presenting low magnetosome content.

Video S3 mbio.03265-25-s0004.avi Representative cryo-electron tomography of an MSR-1 cell grown under microaerobic conditions presenting high magnetosome content.

Video S4 mbio.03265-25-s0005.avi Representative cryo-electron tomography and its 3D volumetric segmentation of an MSR-1 cell grown under microaerobic conditions presenting high magnetosome content.

ASM does not own the copyrights to Supplemental Material that may be linked to, or accessed through, an article. The authors have granted ASM a non-exclusive, world-wide license to publish the Supplemental Material files. Please contact the corresponding author directly for reuse.
