# Supplementary material for: Impact of oxidative stress on Magnetospirillum gryphiswaldense MSR-1 physiology and magnetosome biomineralization at the single-cell level
Source: mBio. 2025 Dec 29;17(2):e03265-25. doi: 10.1128/mbio.03265-25 (PMC12912731; doi:10.1128/mbio.03265-25)
Supplement: Supplemental material — Supplemental figures, tables, and video captions. [file mbio.03265-25-s0001.docx]

**Supplementary information**

**Impact of oxidative stress on *Magnetospirillum gryphiswaldense* MSR-1 physiology and magnetosome biomineralization at the single-cell level**

Marta Masó-Martínez^a,b*^, Julika Radecke^c^, Craig MacGregor-Chatwin^c^, Paul D Topham^b^, Alfred Fernández-Castané^a,b*^

^a^ Energy and Bioproducts Research Institute, Aston University, Birmingham, B4 7ET, UK

^b^ Aston Institute for Membrane Excellence, Aston University, Birmingham, B4 7ET, UK

^c^ Electron Bio-Imaging Centre (eBIC), Diamond Light Source, Harwell Science and Innovation Campus, Didcot OX11, UK

*Corresponding author email: m.masomartinez@aston.ac.uk; a.fernandez-castane1@aston.ac.uk

**Supplementary** **Figures**


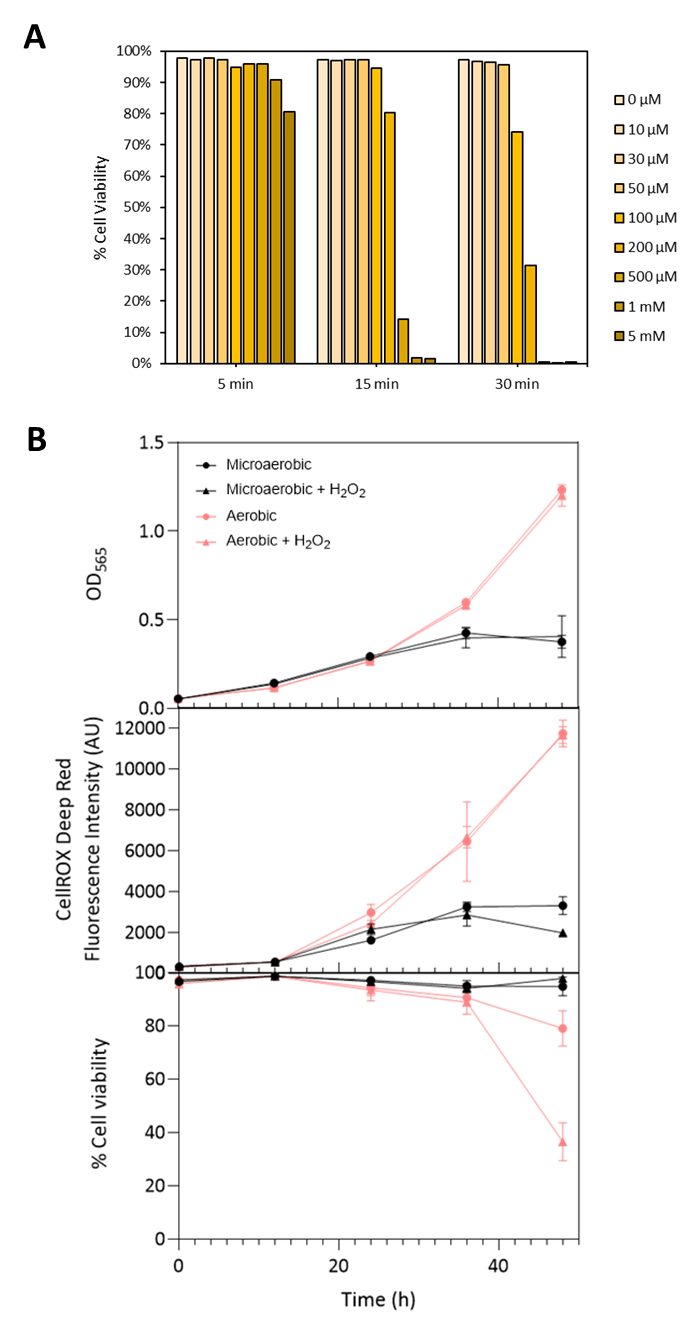


**Figure S1**. (A) MSR-1 H₂O₂ tolerance test results. MSR-1 cells were grown under aerobic conditions and exposed to different H₂O₂ concentrations (0-5 mM) and incubated at room temperature for 5, 15 and 30 min. (B) Effects of 60 µM H₂O₂ on cellular growth, ROS accumulation and cell viability of MSR-1 cells grown under microaerobic and aerobic conditions with or without 60 µM H₂O₂ for 48 h. Cell viability and ROS accumulation were determined using flow cytometry (FCM). 25 000 events were analysed per sample by FCM.

**Supplementary Figure 2**. Extracellular iron citrate concentrations present in FSM media during a period of 60 h in which MSR-1 was grown under different stress conditions. Error bars are standard deviation (n=3).

**Figure S2**. Extracellular iron citrate concentrations present in FSM media during a period of 60 h in which MSR-1 was grown under different stress conditions. Error bars are standard deviation (n=3).


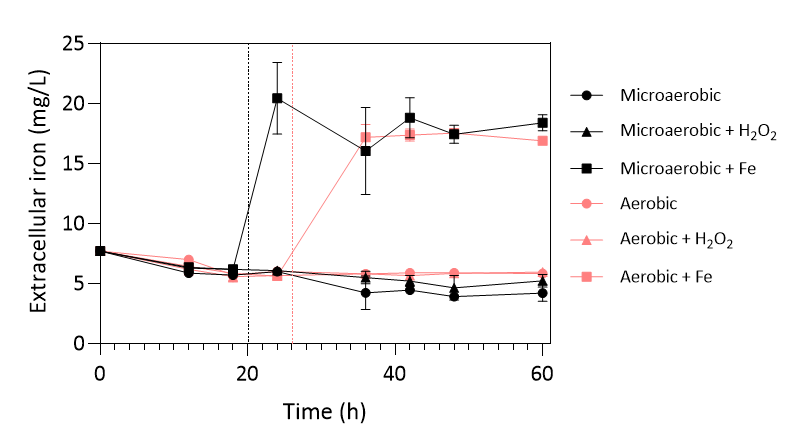

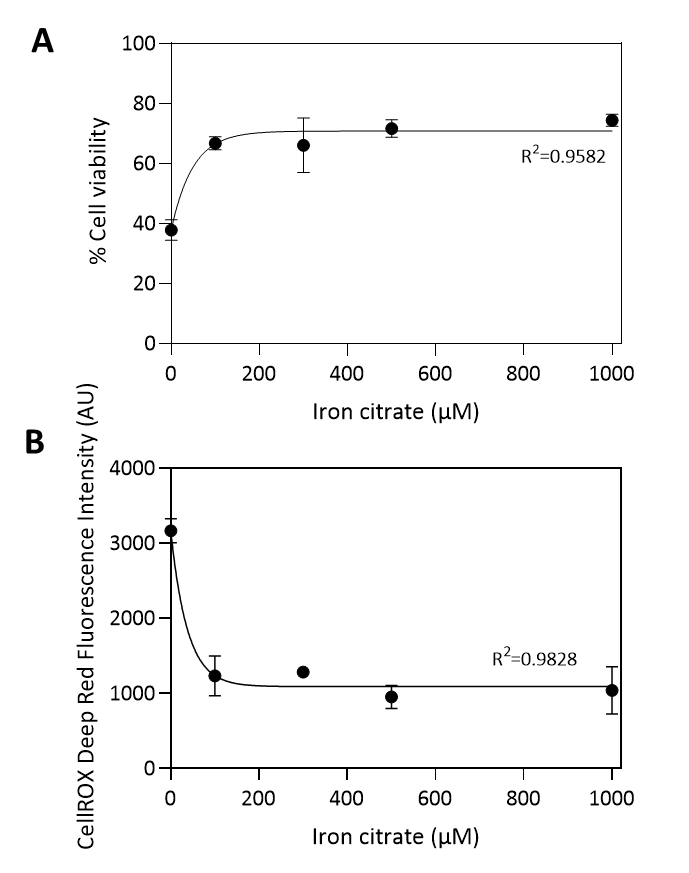


**Figure S3**. (A) Cell viability and (B) ROS accumulation FCM analysis of MSR-1 cells grown at various iron dosages (0-100-300-500-1000 µM iron citrate) under microaerobic conditions. Error bars are standard deviation (n=3). AU= arbitrary units.

**Figure S4.** CryoEM images of MSR-1 cells grown under aerobic (left) and microaerobic (right) conditions. These images show the difference in bacterial cell size when grown under restricted or unrestricted oxygen concentrations.


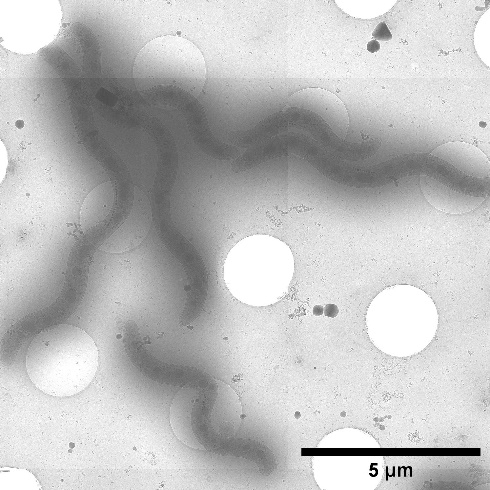

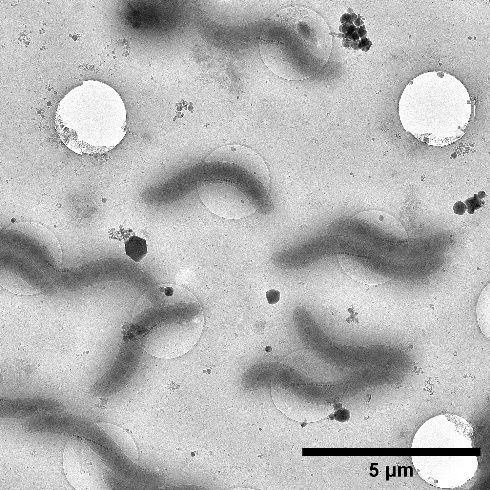


**Aerobic conditions**

**Microaerobic conditions**

**Figure S5.** Flow cytometry analysis of scatter and PHA content of MSR-1 cells exposed to a sudden iron citrate or H₂O₂ pulse mid-growth. (A) Forward scatter (FSC-A), (B) side scatter (SSC-A), and (C) Pyr-546 fluorescence intensity values over time. Vertical lines in graph indicate the addition of iron and H₂O₂ pulses. AU = arbitrary units.


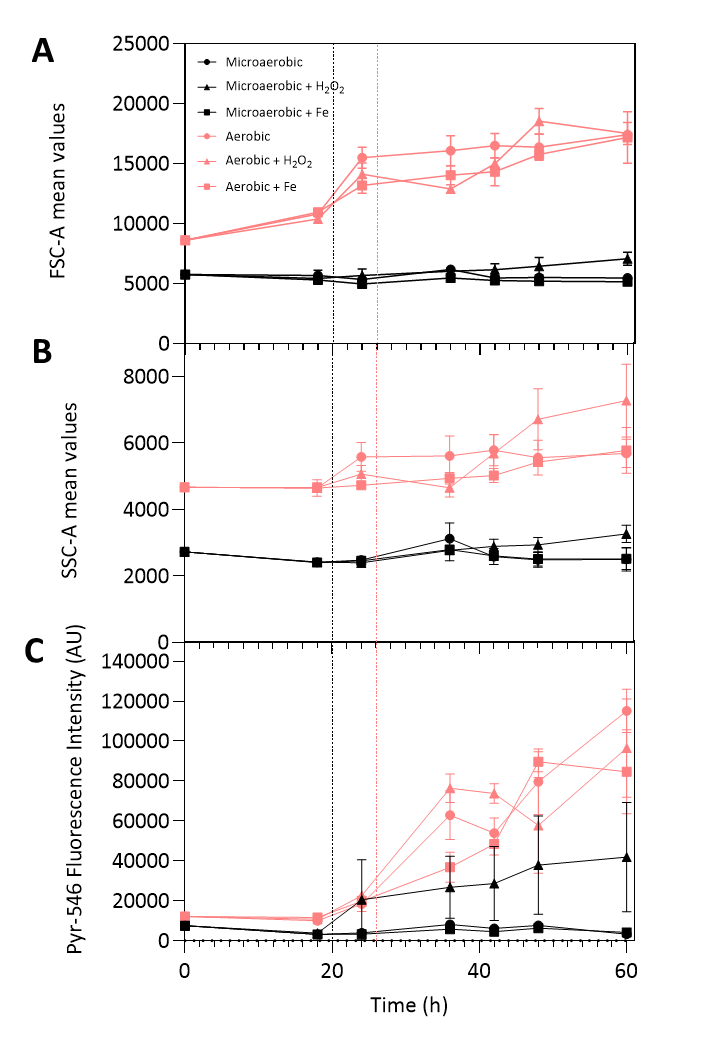


**Figure S6.** Correlation between cell size (FSC-A) and CRDR fluorescence intensity. Each point represents a sample collected at a specific time point measured by flow cytometry. FSC-A= Forward scatter; CRDR= CellROX Deep Red; AU = arbitrary units.


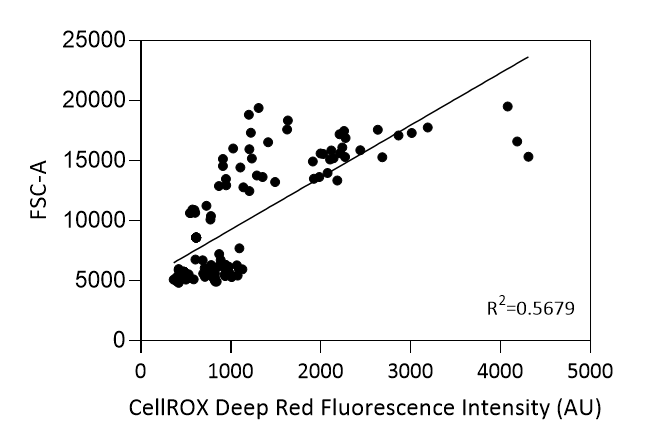

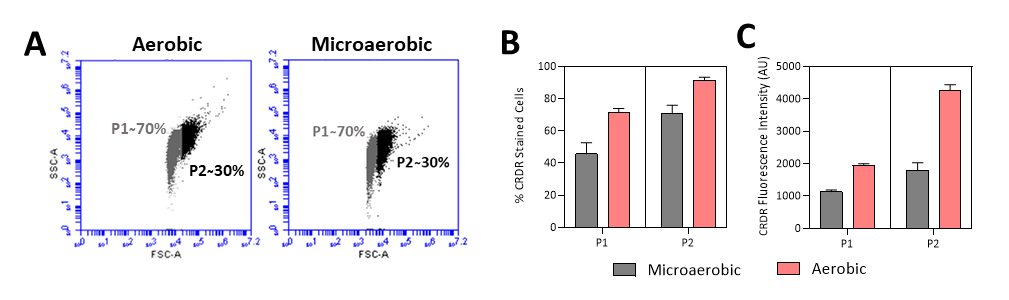


**Figure S7**. (A) Representative forward scatter (FSC-A) *vs* side scatter (SSC-A) dot plot of aerobic and microaerobic MSR-1 cells, in which two distinct populations (P1 and P2) can be distinguished. Comparison between aerobic and microaerobic MSR-1 cells of (B) the percentage of CRDR-stained cells and (C) the fluorescence intensity values of the CRDR-stained cells only of P1 and P2. CRDR = CellROX Deep Red; AU = arbitrary units; P1= population 1; P2 = population 2.

**Supplementary tables**

**Table S1**. One-way ANOVA followed by Tukey’s post-hoc test was performed on MSR-1 cell length. Data represent mean ± SD from 50 bacteria per condition. Significance is indicated with asterisks: * for adjusted p-values between 0.01 and 0.05, ** for p-values between 0.001 and 0.01, *** for p-values between 0.0001 and 0.001, **** for p-values < 0.0001, and ns indicates not significant.

| **ANOVA table** | | **SS** | | **DF** | **MS** | | **F (DFn, DFd)** | | | **P value** |  |
| --- | --- | --- | --- | --- | --- | --- | --- | --- | --- | --- | --- |
| Treatment (between columns) | | 766.9 | | 5 | 153.4 | | F (5, 293) = 37.85 | | | P<0.0001 |  |
| Residual (within columns) | | 1187 | | 293 | 4.052 | |  | | |  |  |
| Total | | 1954 | | 298 |  | |  | | |  |  |
| **Tukey's multiple comparisons test** | **Mean Diff.** | | **99.00% CI of diff.** | | | **Significant?** | | **Summary** | **Adjusted P Value** | | |
| Microaerobic vs. Microaerobic + H_2_O_2_ | 2.383 | | 1.008 to 3.757 | | | Yes | | **** | <0.0001 | | |
| Microaerobic vs. Microaerobic + Fe | 0.1096 | | -1.265 to 1.484 | | | No | | ns | 0.9998 | | |
| Microaerobic vs. Aerobic | -2.244 | | -3.618 to -0.8695 | | | Yes | | **** | <0.0001 | | |
| Microaerobic vs. Aerobic + H_2_O_2_ | 1.604 | | 0.2298 to 2.979 | | | Yes | | ** | 0.0013 | | |
| Microaerobic vs. Aerobic + Fe | -1.438 | | -2.812 to -0.06325 | | | Yes | | ** | 0.0059 | | |
| Microaerobic + H_2_O_2_ vs. Microaerobic + Fe | -2.273 | | -3.641 to -0.9058 | | | Yes | | **** | <0.0001 | | |
| Microaerobic + H_2_O_2_ vs. Aerobic | -4.627 | | -5.994 to -3.259 | | | Yes | | **** | <0.0001 | | |
| Microaerobic + H_2_O_2_ vs. Aerobic + H_2_O_2_ | -0.7786 | | -2.146 to 0.5889 | | | No | | ns | 0.3836 | | |
| Microaerobic + H_2_O_2_ vs. Aerobic + Fe | -3.821 | | -5.188 to -2.453 | | | Yes | | **** | <0.0001 | | |
| Microaerobic + Fe vs. Aerobic | -2.354 | | -3.721 to -0.9860 | | | Yes | | **** | <0.0001 | | |
| Microaerobic + Fe vs. Aerobic + H_2_O_2_ | 1.495 | | 0.1272 to 2.862 | | | Yes | | ** | 0.0033 | | |
| Microaerobic + Fe vs. Aerobic + Fe | -1.547 | | -2.915 to -0.1798 | | | Yes | | ** | 0.002 | | |
| Aerobic vs. Aerobic + H_2_O_2_ | 3.848 | | 2.481 to 5.216 | | | Yes | | **** | <0.0001 | | |
| Aerobic vs. Aerobic + Fe | 0.8062 | | -0.5613 to 2.174 | | | No | | ns | 0.3433 | | |
| Aerobic + H_2_O_2_ vs. Aerobic + Fe | -3.042 | | -4.410 to -1.675 | | | Yes | | **** | <0.0001 | | |

**Table S2**. One-way ANOVA followed by Tukey’s post-hoc test was performed on the percentage of PHA area relative to the total cell area. Data represent mean ± SD from 20 MSR-1 cells per condition. Significance is indicated with asterisks: * for adjusted p-values between 0.01 and 0.05, ** for p-values between 0.001 and 0.01, *** for p-values between 0.0001 and 0.001, **** for p-values < 0.0001, and ns indicates not significant.

| **ANOVA table** | | **SS** | | **DF** | **MS** | **F (DFn, DFd)** | | | **P value** | |  |
| --- | --- | --- | --- | --- | --- | --- | --- | --- | --- | --- | --- |
| Treatment (between columns) | | 0.09549 | | 5 | 0.0191 | F (5, 2994) = 185.1 | | | P<0.0001 | |  |
| Residual (within columns) | | 0.309 | | 2994 | 0.0001 |  | | |  | |  |
| Total | | 0.4045 | | 2999 |  |  | | |  | |  |
| **Tukey's multiple comparisons test** | **Mean Diff.** | | **99.00% CI of diff.** | | | | **Significant?** | **Summary** | | **Adjusted P Value** | |
| Microaerobic vs. Microaerobic + H_2_O_2_ | 0.005252 | | 0.003089 to 0.007415 | | | | Yes | **** | | <0.0001 | |
| Microaerobic vs. Microaerobic + Fe | 0.00046 | | -0.001703 to 0.002623 | | | | No | ns | | 0.9801 | |
| Microaerobic vs. Aerobic | 0.01414 | | 0.01197 to 0.01630 | | | | Yes | **** | | <0.0001 | |
| Microaerobic vs. Aerobic + H_2_O_2_ | 0.005554 | | 0.003391 to 0.007717 | | | | Yes | **** | | <0.0001 | |
| Microaerobic vs. Aerobic + Fe | 0.01367 | | 0.01151 to 0.01584 | | | | Yes | **** | | <0.0001 | |
| Microaerobic + H_2_O_2_ vs. Microaerobic + Fe | -0.004792 | | -0.006955 to -0.002629 | | | | Yes | **** | | <0.0001 | |
| Microaerobic + H_2_O_2_ vs. Aerobic | 0.008886 | | 0.006723 to 0.01105 | | | | Yes | **** | | <0.0001 | |
| Microaerobic + H_2_O_2_ vs. Aerobic + H_2_O_2_ | 0.000302 | | -0.001861 to 0.002465 | | | | No | ns | | 0.9972 | |
| Microaerobic + H_2_O_2_ vs. Aerobic + Fe | 0.008422 | | 0.006259 to 0.01059 | | | | Yes | **** | | <0.0001 | |
| Microaerobic + Fe vs. Aerobic | 0.01368 | | 0.01151 to 0.01584 | | | | Yes | **** | | <0.0001 | |
| Microaerobic + Fe vs. Aerobic + H_2_O_2_ | 0.005094 | | 0.002931 to 0.007257 | | | | Yes | **** | | <0.0001 | |
| Microaerobic + Fe vs. Aerobic + Fe | 0.01321 | | 0.01105 to 0.01538 | | | | Yes | **** | | <0.0001 | |
| Aerobic vs. Aerobic + H_2_O_2_ | -0.008584 | | -0.01075 to -0.006421 | | | | Yes | **** | | <0.0001 | |
| Aerobic vs. Aerobic + Fe | -0.000464 | | -0.002627 to 0.001699 | | | | No | ns | | 0.9793 | |
| Aerobic + H_2_O_2_ vs. Aerobic + Fe | 0.00812 | | 0.005957 to 0.01028 | | | | Yes | **** | | <0.0001 | |

**Table S3**. One-way ANOVA followed by Tukey’s post-hoc test was performed on magnetosome chain length. Data represent mean ± SD from 50 bacteria per condition. Significance is indicated with asterisks: * for adjusted p-values between 0.01 and 0.05, ** for p-values between 0.001 and 0.01, *** for p-values between 0.0001 and 0.001, **** for p-values < 0.0001, and ns indicates not significant.

| **Tukey's multiple comparisons test** | **Mean Diff.** | **99.00% CI of diff.** | **Significant?** | **Summary** | **Adjusted P Value** |
| --- | --- | --- | --- | --- | --- |
| Microaerobic vs. Microaerobic + H_2_O_2_ | 3.831 | -0.4969 to 8.160 | No | * | 0.0338 |
| Microaerobic vs. Microaerobic + Fe | -7.249 | -11.58 to -2.920 | Yes | **** | <0.0001 |
| Microaerobic vs. Aerobic | 12.73 | 8.403 to 17.06 | Yes | **** | <0.0001 |
| Microaerobic vs. Aerobic + H_2_O_2_ | 16.61 | 12.28 to 20.94 | Yes | **** | <0.0001 |
| Microaerobic vs. Aerobic + Fe | 16.19 | 11.86 to 20.52 | Yes | **** | <0.0001 |
| Microaerobic + H_2_O_2_ vs. Microaerobic + Fe | -11.08 | -15.39 to -6.774 | Yes | **** | <0.0001 |
| Microaerobic + H_2_O_2_ vs. Aerobic | 8.9 | 4.594 to 13.21 | Yes | **** | <0.0001 |
| Microaerobic + H_2_O_2_ vs. Aerobic + H_2_O_2_ | 12.78 | 8.474 to 17.09 | Yes | **** | <0.0001 |
| Microaerobic + H_2_O_2_ vs. Aerobic + Fe | 12.36 | 8.054 to 16.67 | Yes | **** | <0.0001 |
| Microaerobic + Fe vs. Aerobic | 19.98 | 15.67 to 24.29 | Yes | **** | <0.0001 |
| Microaerobic + Fe vs. Aerobic + H_2_O_2_ | 23.86 | 19.55 to 28.17 | Yes | **** | <0.0001 |
| Microaerobic + Fe vs. Aerobic + Fe | 23.44 | 19.13 to 27.75 | Yes | **** | <0.0001 |
| Aerobic vs. Aerobic + H_2_O_2_ | 3.88 | -0.4264 to 8.186 | No | * | 0.0289 |
| Aerobic vs. Aerobic + Fe | 3.46 | -0.8464 to 7.766 | No | ns | 0.0726 |
| Aerobic + H_2_O_2_ vs. Aerobic + Fe | -0.42 | -4.726 to 3.886 | No | ns | 0.9995 |

**Table S4**. One-way ANOVA followed by Tukey’s post-hoc test was performed on magnetite size. Data represent mean ± SD from 500 magnetosome crystals per condition. Significance is indicated with asterisks: * for adjusted p-values between 0.01 and 0.05, ** for p-values between 0.001 and 0.01, *** for p-values between 0.0001 and 0.001, **** for p-values < 0.0001, and ns indicates not significant.

| **ANOVA table** | | **SS** | | **DF** | **MS** | **F (DFn, DFd)** | | | **P value** | |  |
| --- | --- | --- | --- | --- | --- | --- | --- | --- | --- | --- | --- |
| Treatment (between columns) | | 4528 | | 5 | 905.7 | F (5, 113) = 33.64 | | | P<0.0001 | |  |
| Residual (within columns) | | 3042 | | 113 | 26.92 |  | | |  | |  |
| Total | | 7570 | | 118 |  |  | | |  | |  |
| **Tukey's multiple comparisons test** | **Mean Diff.** | | **99.00% CI of diff.** | | | | **Significant?** | **Summary** | | **Adjusted P Value** | |
| Microaerobic vs. Microaerobic + H_2_O_2_ | -7.401 | | -13.14 to -1.666 | | | | Yes | *** | | 0.0003 | |
| Microaerobic vs. Microaerobic + Fe | 6.439 | | 0.7040 to 12.17 | | | | Yes | ** | | 0.0024 | |
| Microaerobic vs. Aerobic | -1.781 | | -7.516 to 3.954 | | | | No | ns | | 0.8917 | |
| Microaerobic vs. Aerobic + H_2_O_2_ | -13.38 | | -19.11 to -7.641 | | | | Yes | **** | | <0.0001 | |
| Microaerobic vs. Aerobic + Fe | -2.421 | | -8.156 to 3.314 | | | | No | ns | | 0.6925 | |
| Microaerobic + H_2_O_2_ vs. Microaerobic + Fe | 13.84 | | 8.179 to 19.50 | | | | Yes | **** | | <0.0001 | |
| Microaerobic + H_2_O_2_ vs. Aerobic | 5.62 | | -0.04119 to 11.28 | | | | No | * | | 0.0108 | |
| Microaerobic + H_2_O_2_ vs. Aerobic + H_2_O_2_ | -5.975 | | -11.64 to -0.3138 | | | | Yes | ** | | 0.0054 | |
| Microaerobic + H_2_O_2_ vs. Aerobic + Fe | 4.98 | | -0.6812 to 10.64 | | | | No | * | | 0.0345 | |
| Microaerobic + Fe vs. Aerobic | -8.22 | | -13.88 to -2.559 | | | | Yes | **** | | <0.0001 | |
| Microaerobic + Fe vs. Aerobic + H_2_O_2_ | -19.82 | | -25.48 to -14.15 | | | | Yes | **** | | <0.0001 | |
| Microaerobic + Fe vs. Aerobic + Fe | -8.86 | | -14.52 to -3.199 | | | | Yes | **** | | <0.0001 | |
| Aerobic vs. Aerobic + H_2_O_2_ | -11.6 | | -17.26 to -5.934 | | | | Yes | **** | | <0.0001 | |
| Aerobic vs. Aerobic + Fe | -0.64 | | -6.301 to 5.021 | | | | No | ns | | 0.9988 | |
| Aerobic + H_2_O_2_ vs. Aerobic + Fe | 10.96 | | 5.294 to 16.62 | | | | Yes | **** | | <0.0001 | |

**Supporting Videos descriptions**

**Movie S1**. MSR-1 cryo-electron tomography showing the damaging effects of H2O2-exposition. Scale bar = 1 µm.

**Movie S2**. Representative cryo-electron tomography of MSR-1 cells grown aerobically presenting low magnetosome content. Scale bar = 1 µm.

**Movie S3**. Representative cryo-electron tomography of an MSR-1 cell grown under microaerobic conditions presenting high magnetosome content. Scale bar = 1 µm.

**Movie S4**. Representative cryo-electron tomography and its 3D volumetric segmentation of an MSR-1 cell grown under microaerobic conditions presenting high magnetosome content. Magnetosomes are coloured in blue, PHA granules in green, outer membrane in orange and inner membrane in light orange. Scale bar = 1 µm.
